# An Approach to Cryptography Based on Continuous-Variable Quantum Neural Network

**DOI:** 10.1038/s41598-020-58928-1

**Published:** 2020-02-07

**Authors:** Jinjing Shi, Shuhui Chen, Yuhu Lu, Yanyan Feng, Ronghua Shi, Yuguang Yang, Jian Li

**Affiliations:** 1 School of Computer Science and Engineering, Central South University, Changsha, 410083 China; 2 Faculty of Information Technology, Beijing University of Technology, Beijing, 100124 China; 3 State Key Laboratory of Network and Switching Technology, Beijing University of Posts and Telecommunications, Beijing, 100876 China

**Keywords:** Engineering, Mathematics and computing, Physics

## Abstract

An efficient cryptography scheme is proposed based on continuous-variable quantum neural network (CV-QNN), in which a specified CV-QNN model is introduced for designing the quantum cryptography algorithm. It indicates an approach to design a quantum neural cryptosystem which contains the processes of key generation, encryption and decryption. Security analysis demonstrates that our scheme is security. Several simulation experiments are performed on the Strawberry Fields platform for processing the classical data “Quantum Cryptography” with CV-QNN to describe the feasibility of our method. Three sets of representative experiments are presented and the second experimental results confirm that our scheme can correctly and effectively encrypt and decrypt data with the optimal learning rate 8*e* − 2 regardless of classical or quantum data, and better performance can be achieved with the method of learning rate adaption (where increase factor R_1_ = 2, decrease factor R_2_ = 0.8). Indeed, the scheme with learning rate adaption can shorten the encryption and decryption time according to the simulation results presented in Figure 12. It can be considered as a valid quantum cryptography scheme and has a potential application on quantum devices.

## Introduction

Cryptography is one of the most crucial aspects for cybersecurity and it is becoming increasingly indispensable in information age. In classical cryptosystems, cryptography algorithms are mostly based on classical hard-to-solve problems in number theory. However, the development of quantum computer and quantum algorithms^1,2^, such as Shor’s algorithm^3^, poses an essential threat on the security of cryptosystems based on number theory difficulties (like RSA cryptosystem). Thus the novel post-quantum cryptography^4^ (including quantum cryptography^5–7^) which is secure against both quantum and classical computers is urgently required. Moreover, the typical scheme of quantum cryptography is implemented by combining quantum key distribution with classical “one-time pad” model^8,9^ currently, which can effectively solve the key distribution problem^10^. While there are the problems of high key rate requirements, large key demands and consumptions in practical applications in the “one-time pad” quantum communication system. Therefore, we approach to investigate new quantum cryptography algorithms and protocols that can be implemented based on a more practical model.

several researchers have already combined neural network with classical cryptography for the multivariate structural and nondirectional features of neural network. In 1990, Lauria^11^ firstly introduced the concept of cryptography based on artificial neural network (ANN). Then branches of applications and related works of cryptography with different ANN models were proposed subsequently. Network stochastic synchronization with partial information^12^ and asymptotic, finite-time synchronization for networks with time-varying delays^13^ provide possibilities for mutual learning between neural networks. Synchronization and learning mechanism based on neural network^14^ prove that neural network can be trained to perform encryption and decryption operations, which is similar to the black box computing model in quantum computation^15^. In addition, Sayantica^16^ demonstrated hackers who have computational power polynomial in time cannot be able to invade in the neural network cryptosystem. Thus it provides an opportunity for the combination of quantum computing and neural cryptography^17^.

Quantum neural network^18^ was firstly proposed by Kak and it provided a potential solution to design novel encryption and decryption mechanism with computational efficiency, quantum natural properties, unidirectionality and multivariate structure of ANN. The advantages of quantum neural network in fast learning, improving efficiency of information processing and ensuring itself effectiveness have been highlighted^19–21^. In recent years, discrete-variable quantum neural network (DV-QNN) have been researched and serval practical applications have also been developed^22–25^. General achievable classification scheme based on quantum neural network^26^ and quantum neural network optimization based on variational algorithm^27^ promote the practical progress of quantum neural network. Cryptography system based on DV-QNN^28^ was firstly introduced in 2016, which applied quantum neural network into encryption and decryption area. While a cryptosystem Anh *et al*.^28^ proposed is required to prepare the discrete quantum source and design the gradient descent algorithm corresponding to classical training algorithm, which increased the difficulty of practical implementation of the cryptosystem.

Thus continuous-variable quantum neural network (CV-QNN) model is utilized in this paper to design a more practical quantum cryptography scheme, which can be considered as an approach to quantum neural cryptography (QNC). Gaussian states which are experimental easier-to-achieve resources^29,30^ compared with single photon are utilized instead of discrete-variable quantum states. A specific quantum neural cryptography model is devised based on the general CV-QNN with additional preprocessing and postprocessing. In the preprocessing, legitimate measurement bases (LMB) are introduced to resist information eavesdropping, and the involved quantum nonlinear mapping method allows classical bits to be encoded into quantum states, which increases the types of input information. Mature optimization algorithm Adam^31^ is utilized in the process of training QNC for adjusting weights correctly and efficiently, and the message authentication against message replay is introduced. The experimental results simulated on Strawberry Fields^32^ demonstrate that the scheme can correctly encrypt and decrypt data and the method of learning rate adaption in our paper can accelerate the cryptography algorithm and strengthen the security of the cryptosystem.

## Methods

### Continuous-variable quantum neural network model

According to the structural characteristics of discrete and continuous spectrum of the quantum eigenstates, quantum states can be divided into two categories: discrete variables and continuous variables, and discrete variable quantum information theory has been widely researched. It inspirits the continuous-variable quantum fields including the extension of quantum information communication from finite to infinite dimensions. In continuous-variable fields, information represented by qumodes is carried in the quantum states of bosonic modes, and continuous quadrature amplitudes of the quantized electromagnetic field can be applied to implement quantum state preparation, unitary manipulation and quantum measurement^33,34^. Unlike discrete variable quantum models that perform unitary operations, such as Pauli matrixes, continuous-variable quantum models often utilize Gaussian and non-Gaussian operators^33^ to transform quantum states. For a qumode $\hat{x}$ which can be described with two real-valued variables $(x,p)\in {{\mathbb{R}}}^{2}$, the transformations on phase space with Gaussian operation gates^34^ can be summarized as follows:

1 $$R(\phi ):\left(\begin{array}{c}x\\ p\end{array}\right)\mapsto \left(\begin{array}{cc}cos\phi  & sin\phi \\ -sin\phi  & cos\phi \end{array}\right)\left(\begin{array}{c}x\\ p\end{array}\right),$$

2 $$D(\alpha ):\left(\begin{array}{c}x\\ p\end{array}\right)\mapsto \left(\begin{array}{c}x+Re(\alpha )\\ p+Im(\alpha )\end{array}\right),$$

3 $$S(r):\left(\begin{array}{c}x\\ p\end{array}\right)\mapsto \left(\begin{array}{cc}{e}^{-r} & 0\\ 0 & {e}^{r}\end{array}\right)\left(\begin{array}{c}x\\ p\end{array}\right),$$

4 $$BS(\theta ):\left(\begin{array}{c}{x}_{1}\\ {x}_{2}\\ {p}_{1}\\ {p}_{2}\end{array}\right)\mapsto \left(\begin{array}{llll}cos\theta  & -sin\theta  & 0 & 0\\ sin\theta  & cos\theta  & 0 & 0\\ 0 & 0 & cos\theta  & -sin\theta \\ 0 & 0 & sin\theta  & cos\theta \end{array}\right)\left(\begin{array}{c}{x}_{1}\\ {x}_{2}\\ {p}_{1}\\ {p}_{2}\end{array}\right),$$

where the simplest single mode Gaussian gates $R(\phi )$, $D(\alpha )$, $S(r)$ are *rotation* gate, *displacement* gate and *squeezing* gate respectively, and the (phaseless) *beamsplitter* $BS(\theta )$ indicates the basic two mode Gaussian gate. The ranges for the parameter values are $\phi$, $\theta \in [0,2\pi )$, $\alpha \in {\mathbb{C}}\cong {{\mathbb{R}}}^{2}$, and *r* ≥ 0.

**Figure 1:**
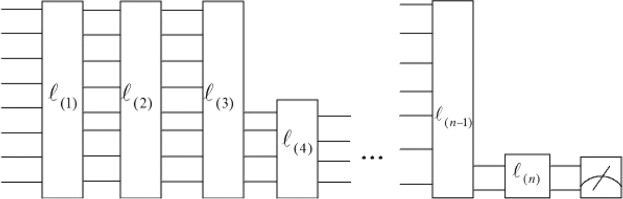
A general continuous-variable quantum neural network model. ${\ell }_{(\iota )}$ for $\iota \in \{1,2,\,\mathrm{..}.,\,n\}$ represents a single layer of quantum neural network. The width of layers can be decreased by tracing out some qumodes or can be increased by increasing some auxiliary qumodes. The output of the last layer can be measured to obtain valued information.

A general CV-QNN model^34^ is presented in Fig. 1. The width of the later layers can be decreased (increased) by tracing out qumodes (increasing ancillary qumodes) and the output of the last layer can be measured to obtain valued information. By the way, classical neural network can be embedded into the general CV-QNN model by fixing gate parameters so that the formalism may not create any superposition or entanglement. In other words, the CV-QNN can deal with classical data, i.e., the input $|c\rangle$ can be created by applying the *displacement* operator *D*(*c*) where *c* is classical data to the vacuum state:

5 $$c\leftrightarrow |c\rangle :\,=|{c}_{1}\rangle \otimes |{c}_{2}\rangle \otimes \cdot \cdot \cdot |{c}_{n}\rangle .$$

In addition, different QNN models, such as recurrent quantum neural network, can be reasonably constructed with the changeable structure in Fig. 1, and a neuron of quantum neural network needs to be specified as well to achieve different functions.

### Training algorithms for quantum neural network

An initial neural network is required to be trained so that it can handle practical problems, such as correctly encrypt and decrypt data or classify images, etc. The methods for training QNN roughly fall into two main categories:

- Optimize neural network parameters with existing quantum algorithms. Such as, utilize the quantum search algorithm to find optimal weights for network^35^.
- Generate quantum training algorithms corresponding to the classical training algorithms to find the optimal value of target function.

Gradient descent belonging to the second category can be applied to quantum computation, which is universal that great quantities of modules on the programming software platform have the ability to automatically compute the optimum gradient. In this scheme, we perform experiments on Strawberry Fields^32^ and adopt Adam algorithm to optimize CV-QNN. Adam algorithm is a stochastic gradient descent algorithm, which is suitable for optimizing quantum neural cryptosystem due to its non-deterministic but optimized output. Specifically, optimizing quantum neural network can be implemented by adjusting parameters of transformation matrices. Take the *rotation* operator $R({\phi }^{\ast })$ as an example, then the following transformation can be derived after training QNN according to Eq. (1).

6 $$R({\phi }^{\ast }):\left(\begin{array}{c}{x}^{\ast }\\ {p}^{\ast }\end{array}\right)\mapsto \left(\begin{array}{cc}cos(\phi +\Delta \phi ) & sin(\phi +\Delta \phi )\\ -sin(\phi +\Delta \phi ) & cos(\phi +\Delta \phi )\end{array}\right)\left(\begin{array}{c}x\\ p\end{array}\right),$$

where ${\phi }^{\ast }=\phi +\Delta \phi$, and $\Delta \phi$ can be determined when the desired target results are achievable. Other transformation matrices in Eqs. (2–4) may have similar evolutions as well.

### Cryptography algorithm based on continuous-variable quantum neural network

Specific model design for cryptography algorithm and the processes of secret-key generation, encryption and decryption with CV-QNN model are provided in this section.

#### Design of CV-QNN for cryptography algorithm

Mathematical isomorphism between the input and output of a neuron verifies that CV-QNN can be utilized to encrypt and decrypt data. According to general function expression of classical neural network *Y* = *f*(W * *X* + *b*), where *W*, *X* and *b* are weight matrix, input vector and bias vector respectively, and *Y* is the output vector of classical neural network. Similarly, we can get theoretical expression between neurons of CV-QNN^34^, i.e.,

7 $${\hat{y}}_{(k)}=|\varphi \left(\mathop{\sum }\limits_{k=1}^{m}\,{W}_{k,j}{\hat{x}}_{(j)}+{\alpha }_{k}\right)\rangle ,$$

where ${W}_{k,j}$ for $k=1,2\ldots m,j=1,2\ldots n$ are unitary operators for transforming the input ${\hat{x}}_{(j)}$ to the output ${\hat{y}}_{(k)}$ with ${\hat{x}}_{(j)}={\int }_{-\infty }^{\infty }\,{x}_{j}|{x}_{j}\rangle \langle {x}_{j}|d{x}_{j}$. By the way, ${\hat{x}}_{(j)}$ represents the *j*th input of the neuron or the *j*th output of a neuron in the last layer, ${\hat{y}}_{(k)}$ represents the *k*th output of the neuron or the *k*th input of a neuron in the next layer. ${\alpha }_{k}$ represents the parameters of *displacement* $D({\alpha }_{(k)})$ and *φ*(·) is nonlinear function. Similarly, mathematical isomorphism between layers of CV-QNN can be summarized as follows:

8 $$\hat{y}=|\phi (W\hat{x}+\alpha )\rangle ,$$

where $$\hat{y}=\left(\begin{array}{c}{\hat{y}}_{(1)}\\ {\hat{y}}_{(2)}\\ \vdots \\ {\hat{y}}_{(m)}\end{array}\right)$$, $$\hat{x}=\left(\begin{array}{c}{\hat{x}}_{(1)}\\ {\hat{x}}_{(2)}\\ \vdots \\ {\hat{x}}_{(m)}\end{array}\right)$$, $$\alpha =\left(\begin{array}{c}{\alpha }_{1}\\ {\alpha }_{2}\\ \vdots \\ {\alpha }_{m}\end{array}\right)$$ and $$W=\left(\begin{array}{llll}{W}_{1,1} & {W}_{1,2} & \ldots  & {W}_{1,n}\\ {W}_{2,1} & {W}_{2,2} & \ldots  & {W}_{2,n}\\ \vdots  & \vdots  & \vdots  & \vdots \\ {W}_{m,1} & {W}_{m,2} & \ldots  & {W}_{m,n}\end{array}\right)$$. In addition, the initial inputs of the network can be easily recovered by taking inverse of unitary matrix, i.e.,

9 $$\hat{x}=|\varphi ({W}^{-1}(\hat{y}-\varphi (\alpha )))\rangle .$$

**Figure 2:**
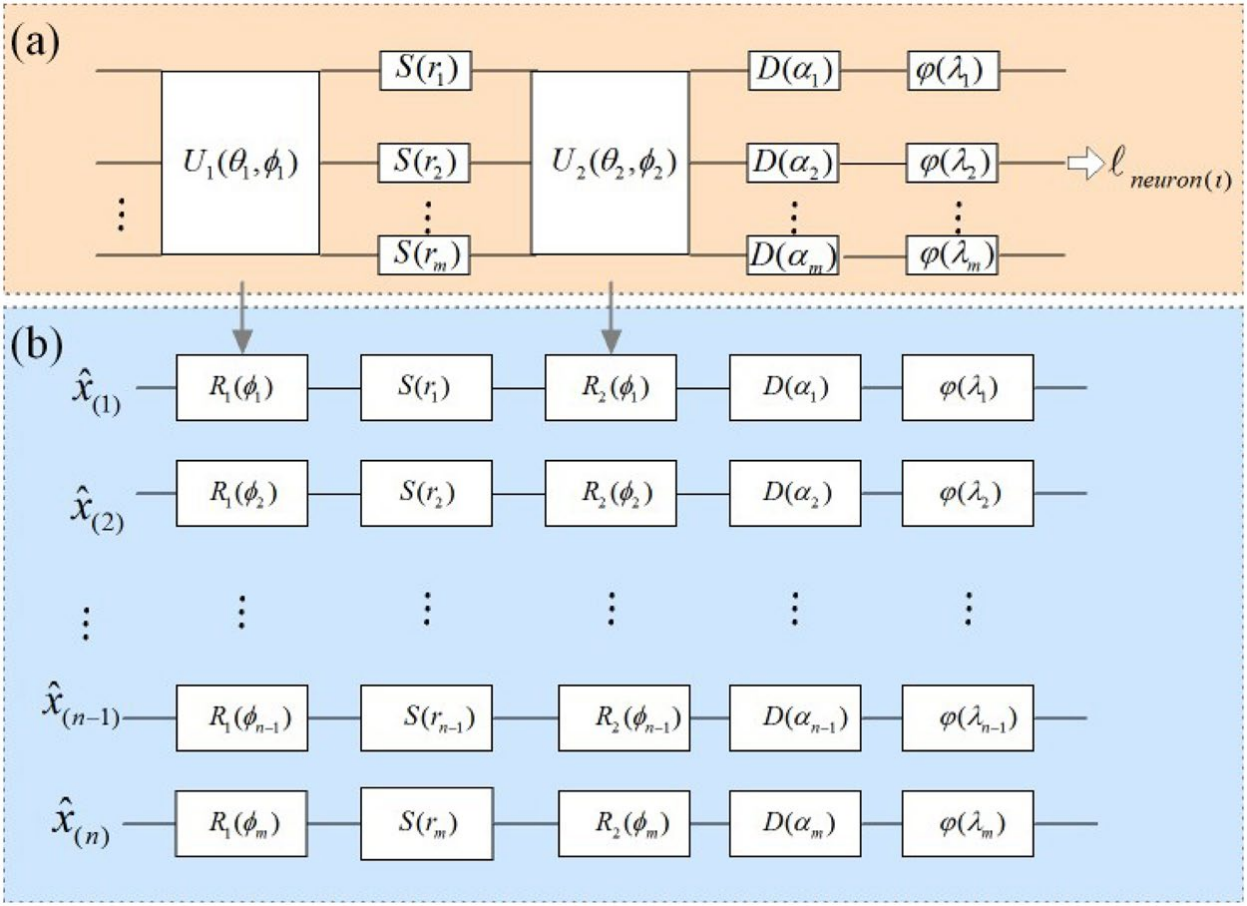
(**a**) The schematic of a general neuron of CV-QNN. (**b**) The schematic of a specific neuron ${\ell }_{neuron(\iota )}$ which includes the first local rotations ${R}_{1}({\phi }_{k})$, local squeeze gates *S*(*r*_*k*_), the second local rotations ${R}_{2}({\phi }_{k})$, local displacements $D({\alpha }_{k})$, and local non-Gaussian gates $\varphi ({\lambda }_{k})$ for $k=1,2\ldots m$. The first four components implement an affine transformation, followed by a final nonlinear transformation.

In order to design the cryptography model effectively and practically to conforms to Eq. (8), Gaussian and non-Gaussian operators are fixed to construct a neuron of quantum neural network. Fig. 2(a) introduces the schematic of general neurons of CV-QNN^34^ corresponding to neurons of the layer ${\ell }_{(\iota )}$, and the schematic of specific neurons for cryptography model is presented in Fig. 2(b) where *rotation* operators ${R}_{1}$ and ${R}_{1}$ take the place of ${U}_{1}$ and ${U}_{2}$ in Fig. 2(a) respectively. Hence, a neuron can be defined as follows:

10 $${\ell }_{neuron(\iota )}\,:\,=\varphi \,\circ \,D\,\circ \,{R}_{2}\,\circ \,S\,\circ \,{R}_{1}.$$

where nonlinear function $\varphi$ can be implemented by non-Gaussian operator Kerr $K(\kappa )=exp(i\kappa {\hat{n}}^{2})$ with Hamiltonian $H=\hat{a}{\hat{a}}^{\dagger }={\hat{n}}^{2}$, $t\in {\mathbb{R}}$ ($\hat{a}$ and ${\hat{a}}^{\dagger }$ are the annihilation and creation operators respectively). It is obvious that $|{W}_{k,j}\rangle :\,={R}_{2}\,\circ \,S\,\circ \,{R}_{1}$ for $k=1,2\ldots m,j=1,2\ldots n$, and $|{W}_{k,j}{\hat{x}}_{(j)}+{\alpha }_{k}\rangle :\,=D\,\circ \,{R}_{2}\,\circ \,S\,\circ \,{R}_{1}$, the phase of Gaussian operations $D$, ${R}_{2}$, $S$, ${R}_{1}$ are just contained in ${W}_{k,j}$ and ${\alpha }_{k}$. While during the process of training CV-QNN, only the weight ${W}_{k,j}$ can be changeable. Hence weight can be regarded as secret key for a quantum neural cryptosystem.

**Figure 3:**
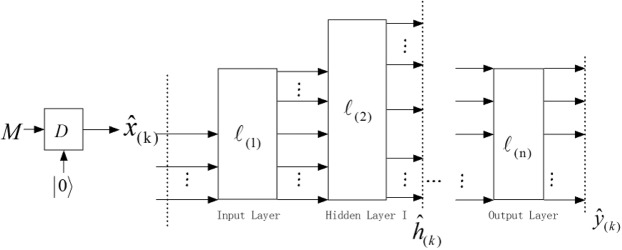
Multilayer CV-QNN for the cryptosystem. The preprocessing is in the left part where $M$ is classical data. The process of encrypting data is in the right part. ${\hat{x}}_{(k)}$ are inputs for the neural network, ${\hat{y}}_{(k)}$ are direct outputs of the last layer, ${\hat{h}}_{(k)}$ represent the outputs of hidden layer. The width of hidden layers can be changeable by tracing some qumodes out or increasing some auxiliary qumodes.

Above discussions demonstrate that quantum neural network can be properly applied as cryptogrsystem with secret key $W$. Thus, a cryptography model can be designed by multi-layer CV-QNN which is presented in Fig. 3 where the inputs ${\hat{x}}_{(k)}$ are transformed by plaintext $M$ according to Eq. (5). The process of ${\hat{x}}_{(k)}$ being computed by CV-QNN can be simply described as $\varphi ({\hat{x}}_{(k)})$. Besides, the CV-QNN has two kinds of outputs. One is ${\hat{h}}_{(k)}$ which are the outputs of hidden layer, the other is ${\hat{y}}_{(k)}$ which are direct outputs of the last layer. By the way, ${\hat{h}}_{(k)}$ can be used to verify the integrity of data, and ${\hat{y}}_{(k)}$ can be utilized to construct cipher block (mentioned in the next section in detail).

#### Key generation

It is well known that the weight may be random before training CV-QNN, thus the quantum neural network is required to be trained with lots of training sets and training algorithm for processing data correctly. During the process of training, the weights *W* in Eq. (8) are updated, i.e., secret keys are generated. In addition, the network architectures, the chosen optimization algorithms and the training sets which are unrevealed, can determine the distributions of weights of hidden layers^36^. In other words, the network architectures *et al* also can be regarded as the keys for quantum neural cryptosystem. Hence, multiple keys are contained in the cryptosystem, so that adversaries are difficult to obtain all configurations above to acquire the secret keys. Moreover, the dimensions of input and output and the hierarchy of hidden layers decide the length and complexity of keys. Thus, valid users can change the length of keys accordingly to satisfy the security of communications^37^.

#### Encryption

If the plaintext $M$ are classical data, then the data are required to be preprocessed into qumodes according to $D(M)|0\rangle \leftrightarrow \hat{x}$ which are mapped to $\hat{x}:\,=\{{\hat{x}}_{(1)},{\hat{x}}_{(2)},\mathrm{..}.,{\hat{x}}_{(m)}\}$ in accordance with the dimension $m$ of the input vector. Therefore the total number of encryptions can be defined as $\lceil \frac{L(M)}{m}\rceil$, where $L(M)$ is the length of $M$. The whole process of encryption can be simply presented as Eq. (11).

11 $${\hat{y}}_{(k)}={\left\{\mathop{\mathop{\otimes }\limits_{k=1}}\limits^{m^{\prime} }\varphi ({\lambda }_{k})\mathop{\mathop{\otimes }\limits_{k=1}}\limits^{m^{\prime} }D({\alpha }_{k})\mathop{\mathop{\otimes }\limits_{k=1}}\limits^{m^{\prime} }{R}_{2}({\phi }_{k})\mathop{\mathop{\otimes }\limits_{k=1}}\limits^{m^{\prime} }S({r}_{k})\mathop{\mathop{\otimes }\limits_{k=1}}\limits^{m^{\prime} }{R}_{1}({\phi }_{k})\right\}}^{n}{\hat{x}}_{(k)}.$$

Dimension *m* can be changed as *m*′ vary in each layer by tracing some qumodes out^34^ or increasing ancillary qumodes, and $n$ represents the size of hidden layers. Inputs ${\hat{x}}_{(k)\{k=1,2,\mathrm{..}.,m\}}$ are processed by the first few neural layers, the last layer ${\ell }_{(n)}$ produces a certain amount of outputs denoted by ${\hat{y}}_{(k)}$. Let the output state of the circuit be $|\psi (x)\rangle$ for the given input $D(M)|0\rangle$, so the expectation value of the quadrature operator $\hat{y}$, or namely the outputs of the neural network, is $\langle \hat{y}\rangle$, i.e.,

12 $$\langle \hat{y}\rangle =(\psi (x),\hat{y}\psi (x))=\langle \psi (x)|\hat{y}|\psi (x)\rangle .$$

Hence the error function or cost function can be indicated as Eq. (13).

13 $${E}_{(k)}={\hat{x}}_{(k)}-\langle \psi ({\hat{x}}_{(k)})|{\hat{y}}_{(k)}|\psi ({\hat{x}}_{(k)})\rangle .$$

**Figure 4:**
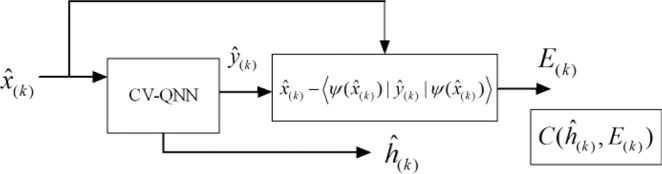
The process of encryption. The CV-QNN can be considered as a black box to generate ${\hat{h}}_{(k)}$ and ${\hat{y}}_{(k)}$ which are target sets. The expected value of ${\hat{y}}_{(k)}$ combines with ${\hat{x}}_{(k)}$ to form error function ${E}_{(k)}$. Then the cipher block $C({\hat{h}}_{(k)},{E}_{(k)})$ is constructed to be sent to the receiver.

The process of encryption is shown in Fig. 4, where the qumodes ${\hat{x}}_{(k)}$ can be input into CV-QNN in batches or once. ${\hat{y}}_{(k)}$ are the final outputs of the neural network which are computed to get ${E}_{(k)}$. ${\hat{h}}_{(k)}$ from the outputs of hidden layer of CV-QNN can be served as the message authentication code (MAC)^37–39^, and then cipher block $C({\hat{h}}_{(k)},{E}_{(k)})$ can be constructed. Apparently, the cryptosystem both implements information encryption and the features of MAC.

#### Decryption

**Figure 5:**
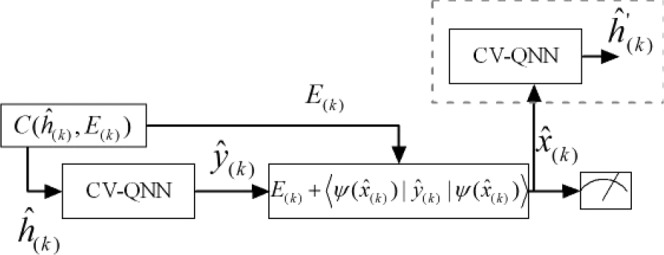
The process of decryption. ${\hat{h}}_{(k)}$ and ${E}_{(k)}$ can be parsed according to cipher block $C({\hat{h}}_{(k)},{E}_{(k)})$. ${\hat{h}}_{(k)}$ is input into the CV-QNN for outputting ${\hat{y}}_{(k)}$, plaintext ${\hat{x}}_{(k)}$ can be achieved with the combination of ${E}_{(k)}$ and $\langle \psi ({\hat{x}}_{(k)})|{\hat{y}}_{(k)}|\psi ({\hat{x}}_{(k)})\rangle$. The process of data verification is shown in the top dotted box, where ${\hat{h}^{\prime} }_{(k)}$ is used to verify the integrity of the data received by the receivers.

The process of decryption is shown in Fig. 5 where the cipher block $C({\hat{h}}_{(k)},{E}_{(k)})$ is parsed into ${\hat{h}}_{(k)}$ and ${E}_{(k)}$. Input ${\hat{h}}_{(k)}$ into the CV-QNN for decryption and then output ${\hat{y}}_{(k)}$. Hence the plaintext ${\hat{x}}_{(k)}$ can be obtained according to Eq. (13). Let the obtained ${\hat{x}}_{(k)}$ be input into the CV-QNN again, then ${\hat{h}^{\prime} }_{(k)}$ can be computed out. Comparing ${\hat{h}^{\prime} }_{(k)}$ with ${\hat{h}}_{(k)}$, we can verify whether the data ${\hat{x}}_{(k)}$ are integrity. In detail, $\langle {\hat{h}^{\prime} }_{(k)}|{\hat{h}}_{(k)}\rangle$ can be derived by means of the swap test^40^. if $\langle {\hat{h}^{\prime} }_{(k)}|{\hat{h}}_{(k)}\rangle \ge 1-\varepsilon$ where $\varepsilon$ is the limitation of fault tolerance, Bob then can accept the integrated ${\hat{x}}_{(k)}$.

**Figure 6:**
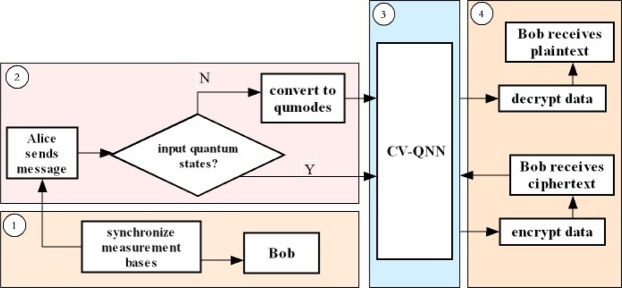
The whole communication stages between sender and recipient, namely Alice and Bob. The first stage is to obtain LMB for Alice and Bob. The second stage is preprocessing for transforming classical data into qumodes. Besides, qumodes should be represented by LMB. The third stage is the preparation of initial keys. The fourth stage is encryption and decryption.

**Figure 7:**
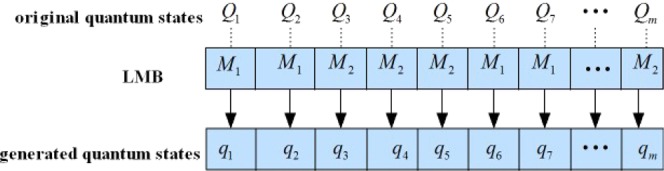
An example that Alice transforms *m* quantum states {*Q*_1_, *Q*_2_, ⋯, *Q*_*m*_} into $\{{q}_{1},{q}_{2},\,\cdot \cdot \cdot ,\,{q}_{m}\}$ with *m* sets of $M{B}^{(A)}$ which are randomly selected from $\{M{B}_{1},M{B}_{2}\}$. It can be seen that serial numbers of $M{B}^{(A)}$ are “1112211...2”.

The whole communication stages between Alice and Bob are illustrated in Fig. 6. Alice and Bob communicate with each other in an identical neural network. The first stage is that Alice and Bob synchronize measurement basis (MB) together (synchronized MB are denoted as LMB). The process of synchronization can be described as following steps: (i) Alice sends quantum states generated by random sets of $M{B}^{(A)}$ to Bob. (ii) Bob measures the quantum states with random sets of $M{B}^{(B)}$ and sends serial numbers of $M{B}^{(B)}$ to Alice. (iii) Alice tells Bob that which serial numbers of $M{B}^{(B)}$ should be reserved so that they can keep the same MB, i.e., $M{B}^{(A)}=M{B}^{(B)}$. Specifically, Alice transforms $m$ quantum states {*Q*_1_, *Q*_2_, …, *Q*_*m*_} into $\{{q}_{1},{q}_{2},\cdot \cdot \cdot ,{q}_{m}\}$ with $m$ sets of MB denoted by $M{B}^{(A)}$ which are randomly selected from $\{M{B}_{1},M{B}_{2}\}$ (an example can be seen in Fig. 7). Then Alice sends these quantum states ${q}_{k}$ for $k=\{1,2..,m\}$ to Bob. Bob measures them with $m$ sets of MB denoted by $M{B}^{(B)}$ which are randomly selected from $\{M{B}_{1},M{B}_{2}\}$ as well. Then he sends $m$ serial numbers of $M{B}^{(B)}$ to Alice (the serial number of $M{B}_{i}$ is denoted by $i$ for $i=\{1,2\}$). Alice will inform Bob that which serial number should be reserved. Finally, Alice and Bob both can keep the same MB, i.e, the process of synchronizing MB is completed. The second stage is that $M$ are required to be preprocessed to derive ${\hat{x}}_{(k^{\prime} )}$ which should be represented as ${\hat{x}}_{(k)}$ with LMB, and one or more ${\hat{x}}_{(k^{\prime} )}$ can be transformed by a set of LMB. The third stage is that the neural network is required to be trained for correctly performing encryption and decryption. Finally, Alice sends each block $C({\hat{h}}_{(k)},{E}_{(k)})$ to Bob in the dedicated communication channel. Bob receives these cipher blocks and sends them to the same neural network for decryption.

### Security and performance analysis

With respect to the cryptography algorithm based on CV-QNN, the following types of resistance are introduced in this section to discuss the security and performance.

#### Resistance of attacking on cipher

Currently there are two main attack ways on ciphertext in communications: ciphertext eavesdropping and message replay.

**Figure 8:**
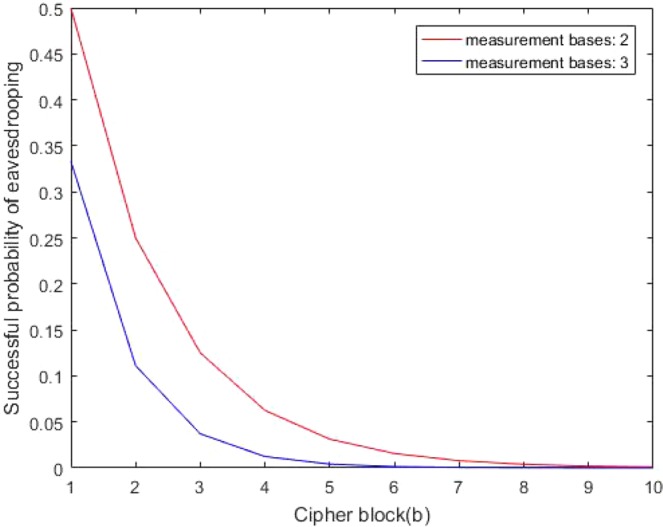
The success probability of cipher eavesdropping for an attacker. When the sets of LMB is 2 and the number of cipher blocks is greater than 10, the success probability of intercepting cipher tends to 0. For the situation that when the sets of LMB is 3 and the number of cipher blocks is just larger than 6, the success probability of cipher eavesdropping is 0.

For the ciphertext eavesdropping attack, the adversary Eve, cannot eavesdrop on correct cipher without the corresponding LMB in the scheme. The number of cipher block and that of LMB is denoted as *a*, *b*, respectively, so the success probability of eavesdropping on cipher is ${(\frac{1}{b})}^{a}$. Fig. 8 demonstrates that the more LMB and cipher blocks can reduce the probability of successfully intercepting cipher. In general communications, just two sets of LMB can contribute to high security. For the message replay attack, assume that Eve wants to cheat the receiver with the prepared quantum states instead of real cipher and she sends the fake cipher to receiver. Specifically, Eve changes ${\hat{h}}_{(k)}$ and/or ${E}_{(k)}$, and sends them to Bob for the purpose of message replay. Then Bob decrypts and gets data ${\hat{x}^{\prime} }_{(k)}$, meanwhile ${\hat{x}^{\prime} }_{(k)}$ are used as the inputs of the neural network to get ${\hat{h}^{\prime} }_{(k)}$. According to the comparison between ${\hat{h}}_{(k)}$ and ${\hat{h}^{\prime} }_{(k)}$, Bob can decide whether to halt this communication or not. Even when a powerful adversary wants to choose the ciphertext $C^{\prime} \{{\hat{h}^{\prime} }_{(k)},{E^{\prime} }_{(k)}\}$ to just succeed in passing the whole verification of MAC, she should change ${\hat{h}}_{(k)}$ and ${E}_{(k)}$ reasonably. Moreover, operations with exponential complexity O$({2}^{2n})$ are required for replaying the $n$-bit cipher. Therefore, the encrypted information cannot be eavesdropped for the attacker lacking corresponding LMB and cipher replay attack cannot be successful for required exponential difficulties to pass the whole MAC. These small probability events of successfully attacking cipher make the scheme achieve high security. This kind of attack is more impossible for CV-QNN with continuous variables, because the attacker cannot know the continuous cipher with brute force. It is also impossible that the invader wants to synchronize an unknown neural network to crack cipher unless he knows the structure of the neural network very clearly^41^. Thus the scheme can resist cipher attack and ensure the security of the proposed cryptography algorithm to the maximum extent.

#### Resistance of system forgery attack

Refer to the situation that the private key is static during the process of an encryption, the cryptanalyst can analyze the key by intercepting numerous of plaintexts with corresponding and available ciphers even in the classical extensive neural network cryptosystem. For simulating a neural network similar to the cryptosystem, the attacker can train a new neural network with the intercepted data and compare the outputs of network with available ciphertext to adjust train algorithm, network architecture etc. to obtain plaintext directly. Furthermore, it is a non-negligible attack for synchronizing network cryptosystems^42^.

Suppose that a hacker can copy the intercepted quantum plain and corresponding cipher to construct a similar cryptography model, which seems to be a threat for our scheme, and it is worth considering. The neural network can keep instable so that the generated cipher can be chaotic and unpredictable for resisting the attack. Similar to TCP congestion control mechanism, learning rate adaption which can adjust the learning rate during the process of encryption contributes to solve the problem^37^. Define a parameter $\xi \in {\mathbb{R}}$ and compare $\xi$ with the value of loss function ${E}_{(k)}$ to control learning rate $\eta$. When $\xi$ is less than ${E}_{(k)}$, learning rate is increased (i.e., $\eta$ multiplied by the increase factor ${R}_{1}$ in Table 1), otherwise reduced (i.e., $\eta$ multiplied by the decrease factor ${R}_{2}$). The instable neural network which can generate chaotic cipher is impossible to be successfully simulated by any hacker who cannot find the laws of encryption. In addition, each plaintext block is encrypted with a pair of corresponding secret keys denoted by ${\tau }_{{k}^{^{\prime\prime} }}$ where $k^{\prime\prime} =1,\,\mathrm{2..}.,\,\lceil \frac{L(M)}{m}\rceil$, the total length of the keys should be the sum of ${\tau }_{{k}^{^{\prime\prime} }}$. According to Eq. (11), the composition of key ${K}_{all}$ can be expressed as

14 $${K}_{all}=\mathop{\prod }\limits_{k^{\prime\prime} =1}^{\lceil \frac{L(M)}{m}\rceil }\,{\left\{\mathop{\sum }\limits_{k^{\prime} =1}^{n}\mathop{\sum }\limits_{k=1}^{m^{\prime} }({\phi }_{k^{\prime} k}^{1}+{r}_{k^{\prime} k}+{\phi }_{k^{\prime} k}^{2}+{\alpha }_{k^{\prime} k}+{\lambda }_{k^{\prime} k})\right\}}^{k^{\prime\prime} },$$

where ${\phi }_{k^{\prime} k}^{1}$ and ${\phi }_{k^{\prime} k}^{2}$are the phase of *rotation* ${R}_{1}$ and ${R}_{2}$ respectively, and “Π” is just the stitching of the total keys. It is apparent that ${K}_{all}$ is hard to be speculated because $\{{\phi }_{k^{\prime} k}^{1},{\phi }_{k^{\prime} k}^{2}\}\in {\mathbb{C}}$. Hence the system can strongly resist the attack on keys, i.e., when messages are encrypted by the neural network, the invader has no way to attack the algorithm or keys regardless of brute force.

**Table 1:** Configuration parameters for the first, second, and  third experiments.

Experiment	Hidden layers	Learning rate	Iterations	Learning rate adaption
1	6	8e − 2	500	×
2	6	8e − 2	500	Control value *ξ* = 0.04 Increase factor R_1_ = 2 Decrease factor R_2_ = 0.8
3	6	>2.0	500	Control value *ξ* = 0.04 Increase factor R_1 _ = 2 Decrease factor R_2_ = 0.8

#### Resistance of the chosen-plaintext attack

The attacker may disguise himself as the sender, then he sends parts of information to the recipient and intercepts ciphers by capturing packets. In this way, the attacker may guess parts or even all keys, or the operating mechanism of the cryptosystem with a certain probability. Let $\kappa$ be a channel composed of plaintext and ciphertext blocks, and private keys, i.e., $\kappa =\{({T}_{plain},{T}_{cipher}),{K}_{prvite}\}$, where ${T}_{plain},{T}_{cipher},{K}_{prvite}$ represent the plaintext, ciphertext and private keys, respectively. The probability of invaders getting ${K}_{prvite}$ using blocks $({T}_{plain},{T}_{cipher})$ is very low for the keys consisting of multiple and continuous parameters as shown in Eq. (14) and secretly preserved ${K}_{prvite}$, especially for quantum information. Due to the fact that under the same conditions, quantum ciphertext ambiguity is higher than classical ciphertext’s^43^ and LMB introduced in the scheme reduces the success probability of chosen-plain attack since correct packets are hard to be captured. So the success probability of the chosen-plaintext attack can be 0, i.e., $p({K}_{prvite}|({T}_{plain},{T}_{cipher}))=0$ when certain security conditions are met, such as increasing the sets of LMB. Just as Fig. 8 shows when the sets of LMB is 2, the success probability of the attacker’s eavesdropping on the correct cipher is 0 only with a few cipher blocks. The mutual information between $({T}_{plain},{T}_{cipher})$ and ${K}_{prvite}$ can be expressed as follows:

15 $$\begin{array}{rcl}I(({T}_{plain},{T}_{cipher}),{K}_{prvite}) & = & \sum p(({T}_{plain},{T}_{cipher}),{K}_{prvite})\\  &  & \times \,\log \,\frac{p({K}_{prvite}|({T}_{plain},{T}_{cipher}))}{p({K}_{prvite})}\\  & = & \sum p({T}_{plain},{T}_{cipher})\,\ast \,p({K}_{prvite}|({T}_{plain},{T}_{cipher}))\\  &  & \times \,\log \,\frac{p({K}_{prvite}|({T}_{plain},{T}_{cipher}))}{p({K}_{prvite})}\\  & = & 0\end{array}$$

$I(({T}_{plain},{T}_{cipher}),{K}_{prvite})=0$ indicates that $\kappa$ is perfect and confidential. Hence the scheme can resist the chosen-plaintext attack.

#### Performance analysis

Due to quantum properties, more classical information can be encoded into multiple degrees of freedom of a quantum state. Hence quantum neural network can carry more information than classical cryptosystem. For the sake of simplicity, classical information and quantum states are one-to-one mapping in our scheme. Compared to the cryptosystem which always requires a new private key for “one-time pad” resulting in increasing the communication time, the cryptography algorithm based on CV-QNN has an effective performance with parallel computational power^44^ and high key utilization. Define the total number of neurons as *mn* where *m* is the number of neurons per neural layer and $n$ is the number of neural layers, the number of average operators in a neuron as ${O}_{p}$. The minimum key utilization ratio can be expressed as *μ*.

16 $$\mu =\frac{L(M)}{mn\,\ast \,{O}_{p}}.$$

**Figure 9:**
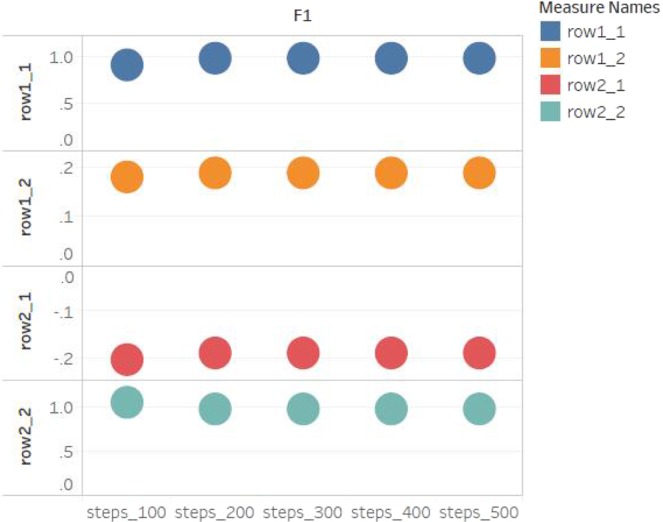
The weights change at different steps. It shows a weight matrix which contains the weights between the 7th layer and the 8th layer of neural network every 100 steps. Different colored circles mark different training steps. Each row represents the element of weight matrix, such as “row1_1” and “row2_1” are the value of the first column of the first row of the matrix and the value of the first column of the second row of the weight matrix respectively. The weights gradually change slowly as the encryption times increase, i.e. the weights become convergent.

With the assistance of learning process of quantum neural network, as the number of encryption increases, the changeable number of weights may slowly decreases. It means that the neural network converges and encrypts faster, especially when correlations are existed between plain. In the Fig. 9, the weight changes at different steps are shown, and the all configuration parameters are from the fourth simulation experiment in subsection “Simulation” of the paper. We can see that from the 100th step to the 500th step, the weight gradually converges, i.e., ${O}_{p}$ becomes small. Particularly, the value of ${O}_{p}$ reduces, and the key utilization $\mu$ increases. Hence compared with other cryptography models which are not based on neural network, quantum neural network uses less keys to encrypt more data.

## Results and Discussion

A CV-QNN model is designed to construct a cryptosystem for encryption and decryption, which is characterized by quantum properties and data processing parallelism of neural network. The multiple and continuous variables, such as phase parameters of *rotation* operation, make the system difficult to be cracked by attackers. Moreover, the additional key negotiation process is not required since the learning process of CV-QNN for encryption and decryption can generate keys. Thus, it is more efficient than other cryptography systems that require key negotiation. The capability of LMB is introduced in the pre-process, which can solve the problem of cipher eavesdropping during the process of communications, though it may increase overheads. Cryptosystem based on ANN is mostly threaten if attackers capture amount of information to simulate a similar neural network to process data. Hence, the analogical method of “TCP congestion control” is applied to keep the network instable for resiting system forgery attacking. The simulated encrypted results demonstrate the security can be improved by adapting parameters (the depth, the learning rate and so on), and the decrypted results show that the original plain can be derived without any error.

### Simulation

Simulation results are presented with the continuous variable quantum simulation platform, named Strawberry Fields^32^ to validate the feasibility of the scheme. The simulated neural network consists of 8 layers, the $cutof{f}_{dim}$ which is Hilbert space truncation dimension is 2. Several experimental simulations are done with different learning rate, and three representative groups of experiments are selected to explore the specific cryptography task. In Table 1, *ξ* is the control value to adapt learning rate for keeping instability of the neural network, and a optimal learning rate is 8e − 2 for the experiments. Training algorithm is Adam which is an automatic optimization algorithm on the simulation platform. It is worth mentioned that the quantum neural network can accept both quantum information and classical information, and during the processes of experimental simulations, classical plain “Quantum Cryptography” is preprocessed into 139-bit binary string, which is taken as an example to be the input of CV-QNN.

**Figure 10:**
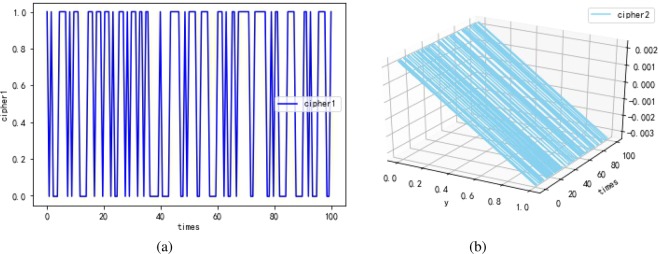
The first experimental results with optimal learning rate ($\eta =8e-2$) and without learning rate adaption. (**a**) The cipher1 from the penultimate output ${\hat{h}}_{(k)}$ of the neural network, and that the cipher1 are just similar to plaintext indicates the quantum neural network can correctly encrypt data well. (**b**) The cipher2 from the error function ${E}_{(k)}$, and times represents the density scale of displayed data.

**Figure 11:**
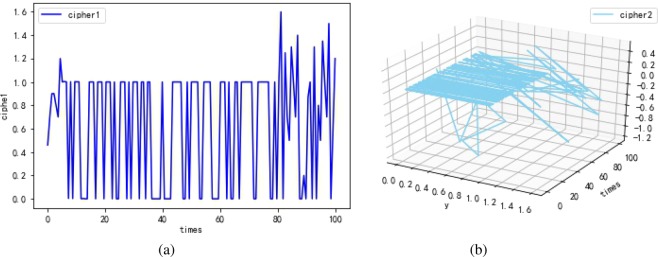
The second experimental results with optimal learning rate ($\eta =8e-2$) and learning rate adaption. (**a**) The cipher1. (**b**) The cipher2. cipher1 gradually approximates plain during the process of encryption. At about 80 times, chaotic cipher1 starts to be formed with the addition of the method of learning rate adaption. Similarly, cipher2 closes to 0 with maximum error rate 0.3% after 20 times and becomes chaotic after 80 times.

**Figure 12:**
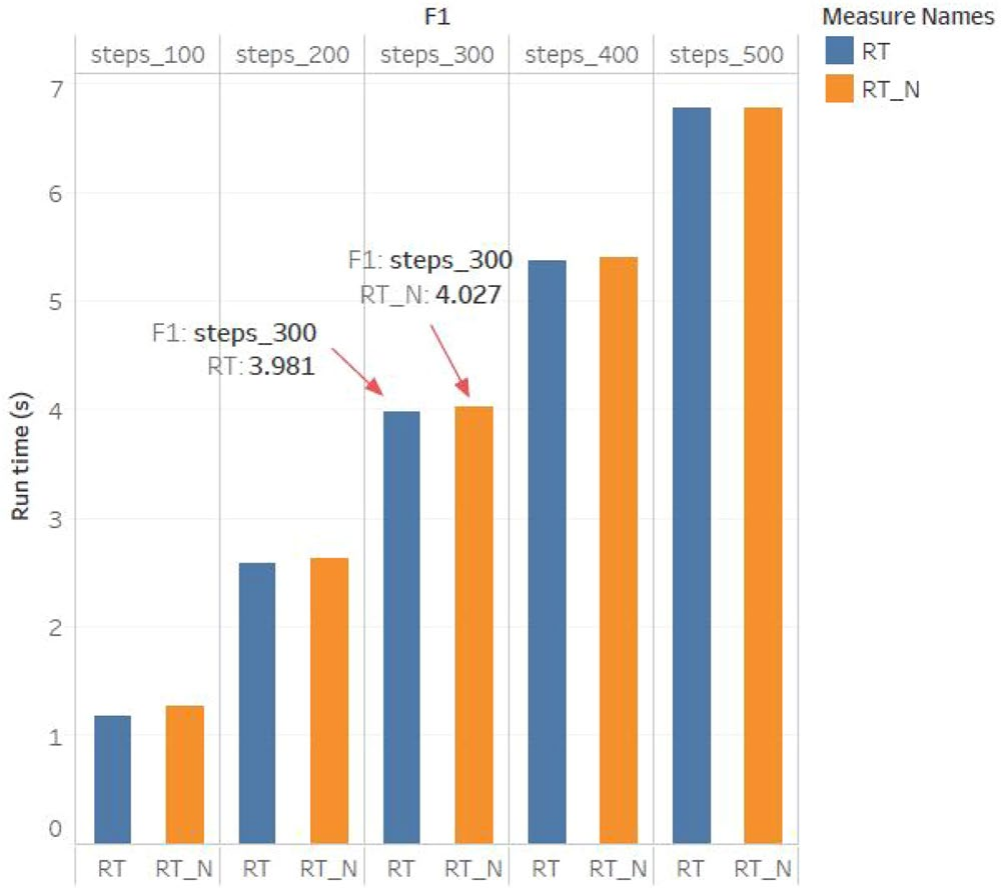
The comparison results between “run time with learning rate adaption” (RT) and “run time without learning rate adaption” (RT-N), or the first experiment and the second experiment where the dominant frequency of running CPU is 3.70 GHz. It can be seen that from the 100th steps to 500th steps, the RT is always less than RT-N. For example, the RT is less than RT-N by around  0.1 s in the 300th steps, which demonstrates that the method of introducing learning rate adaption can accelerate the process of encryption.

**Figure 13:**
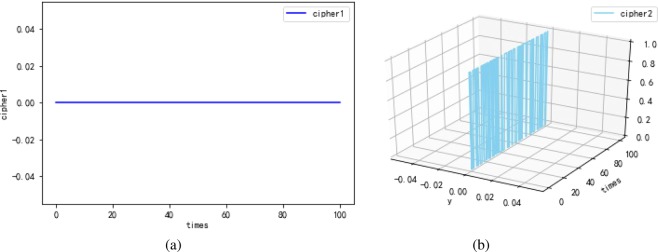
The third experimental results with high learning rate (such as *η* = 3.0) and learning rate adaption. (**a**) The cipher1. (**b**) The cipher2. The values of cipher1 are all 0, which cannot correctly present cipher effects and indicates that the outputs of CV-QNN are too divergent when the learning rate is high.

The first experimental results are shown in Fig. 10. Cipher1 ${\hat{h}}_{(k)}$ (shown in Fig. 10(a)) is the output of penultimate layer of the neural network. Cipher2 *E*_(*k*)_ (shown in Fig. 10(b)) is the 2-dimensional function between input and output, *times* represents the density scale of displayed data. Note that the maximum error rate between ${\hat{x}}_{(k)}$ and ${\hat{y}}_{(k)}$ is only 0.3% according to Fig. 10(b), which verifies that the quantum neural network can correctly encrypt data. Despite cipher1 approximates to plain, attackers are difficult in stealing the all correct cipher by means of intercepting information for the existence of the LMB known only by the sender and receiver. Consider that the static secret keys may expose a quantum neural cryptosystem to system forgery attack. Hence in the second experiment shown in Fig. 11, we try to introduce the solution of “TCP congestion” to keep the neural network instable resisting the attack. To be specific, the neural network should be trained during the process of encryption, when the neural network tends to be stable, the method of learning rate adaption is involved to acquire chaotic cipher. In Fig. 11(a), cipher1 obviously approximates plain ${\hat{x}}_{(k)}$ after 20 times. At about 80 times, the method of learning rate adaption is utilized and then unpredicted ciper1 is generated. Similarly, chaotic cipher2 shown in Fig. 11(b) also can be obtained. The presentation of Fig. 11 demonstrates that the learning rate adaption can improve the security indeed and can reduce the time of encryption process (seen in Fig. 12). The third experimental results are used to analyze the relation between the learning rate and security, and we find that a overlarge learning rate cannot correctly present cipher effects. In Fig. 13, when the learning rate is large (e.g., greater than 2.0 referring to Table 1), the cipher1 ${\hat{h}}_{(k)}$: = 0 (shown in Fig. 13(a)) and cipher2 ${E}_{(k)}\,:={\hat{x}}_{(k)}$ (shown in Fig. 13(b)) which are insensitive to plaintext and cannot provide any information for decryption.

In these experiments, if the attacker wants to intercept correct cipher1 and cipher2, due to the fact that he cannot have a corresponding quantum neural network cryptosystem and LMB for decryption, the violent solving must be his optimal weapon^45^. Thus he needs to try both 2^139^ operators to guess ${\hat{h}}_{(k)}$ and *E*_(*k*)_, and he expects to match ${\hat{h}}_{(k)}$ and *E*_(*k*)_ for 2^139^ * 2^139^ times as well for achieving plaintext. Finally, the attacker needs to try *Ts* times to crack ciphertext, and the probability of correctly guessing cipher is ${(\frac{1}{2})}^{139}{(\frac{1}{2})}^{139}$.

17 $$Ts={2}^{139}\ast {2}^{139}+{2}^{139}+{2}^{139}.$$

**Figure 14:**
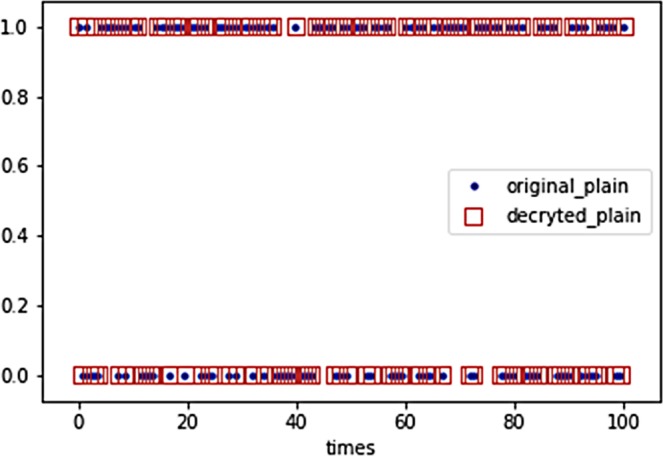
Original plain (described by blue dot) and decrypted plain (described by red square). Decrypted plain is exactly same as original plain in the figure indicating that the CV-QNN is trained well and can decrypt data without any error.

Thus the encrypted classical information with our CV-QNN is intractable to be cracked according to above discussions. For the other situation, when the inputs of CV-QNN are continuous-variable quantum states information, the theoretically unconditional security can be derived for the quantum characters, the continuities of continuous-variable quantum states and the private key. Hence the security of our system can be ensured regardless of the classical information or quantum states. Besides, a decrypted simulation with configuration parameters of the second experiment except for the method of learning rate adaption shows in Fig. 14, where input plaint and decrypted plain are perfectly matched, which demonstrates that constructing a cryptosystem with CV-QNN is effective.

## Conclusions

An available and secure cryptography algorithm has been proposed, in which an extended cryptography model based on CV-QNN is utilized to encrypt and decrypt data. Security and performance analysis shows that the cryptography algorithm can resist cipher eavesdropping, message replay, system forgery attack and the chosen-plaintext attack to guarantee information security and speed up encryption process simultaneously. Moreover, the algorithm inherits the merits of quantum properties, and the experiments results simulated on Strawberry Field platform show that the scheme can correctly encrypt and decrypt data effectively including classical or quantum data. It indicates the first attempt for combining CV-QNN with quantum cryptography, and inspires more potential applications of quantum neural network on quantum devices, such as quantum key distribution (QKD) which can be implemented by the synchronization of QNN.
